# Primary Neuroendocrine Tumor of the Testis Associated With Cardiac Symptoms: A Case Report and Literature Review

**DOI:** 10.7759/cureus.59955

**Published:** 2024-05-09

**Authors:** Eyad A Domlo, Furat A Almayouf, Shatha M Sulaiman, Abdulaziz A Alluhayb, Assem S Alrumeh

**Affiliations:** 1 Pathology Department, King Faisal Specialist Hospital and Research Centre, Riyadh, SAU; 2 Pathology Department, King Fahad Specialist Hospital, Buraydah, SAU; 3 Pathology Department, Ministry of Health, Riyadh, SAU; 4 Emergency Department, Prince Sultan Military Medical City, Riyadh, SAU; 5 Laboratory Department, Prince Sultan Military Medical City, Riyadh, SAU

**Keywords:** cardiac symptoms, orchidectomy, carcinoid syndrome, scrotal mass, testis, well-differentiated neuroendocrine tumor

## Abstract

Well-differentiated neuroendocrine tumors of the testis are exceedingly rare. Here, we report the case of a 47-year-old male patient complaining of cardiac symptoms with a right testicular mass. A right radical orchiectomy was performed. The histopathological findings showed a well-differentiated neuroendocrine tumor with positive synaptophysin and chromogranin A immunostains.

## Introduction

Testicular neuroendocrine tumors of the testis are exceedingly rare neoplasms, accounting for approximately 0.23% of all testicular neoplasms [1]. Primary testicular neuroendocrine tumors may be an incidental finding or can present with scrotal swelling, undescended testis, and carcinoid syndrome [2]. Fewer than 8% of testicular neuroendocrine tumors present with carcinoid syndrome [2]. Here, we report a case of a well-differentiated neuroendocrine tumor of the testis and describe the clinical and histological features of this tumor.

## Case presentation

A 47-year-old male, a known case of type 2 diabetes mellitus, hypertension, and right-side heart failure (due to severe tricuspid regurgitation), was admitted to the emergency department complaining of severe shortness of breath, cough, abdominal distention, and bilateral lower limb swelling for the previous two weeks. There was a history of a painless swelling mass in his right testis for one year.

**Figure 1:**
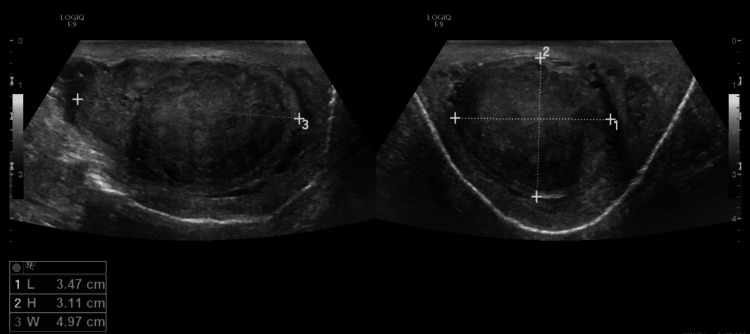
Scrotal ultrasound revealing an intratesticular heterogeneous hypoechoic lesion measuring 3 × 3.6 × 3.4 cm.

Upon physical examination, the patient appeared tachypneic, with abdominal distention, bilateral lower limb edema, and painless swelling of the right testis. Scrotal ultrasound revealed an intratesticular heterogeneous hypoechoic lesion measuring 3 × 3.6 × 3.4 cm (Figure 1). Laboratory investigations showed a high chromogranin A level (5,250 µg/L) and 24-hour urinary 5-hydroxyindoleacetic acid (5-HIAA) level of 11.8 mg/24 hours with normal alpha-fetoprotein and beta-human chorionic gonadotropin (β-HCG) levels.

A right radical orchiectomy was performed. A gross examination of the specimen revealed a well-circumscribed mass measuring 3.5 cm in its greatest dimension and limited to the testis. The epididymis and spermatic cord were unremarkable. The mass was submitted entirely for histologic examination.

**Figure 2:**
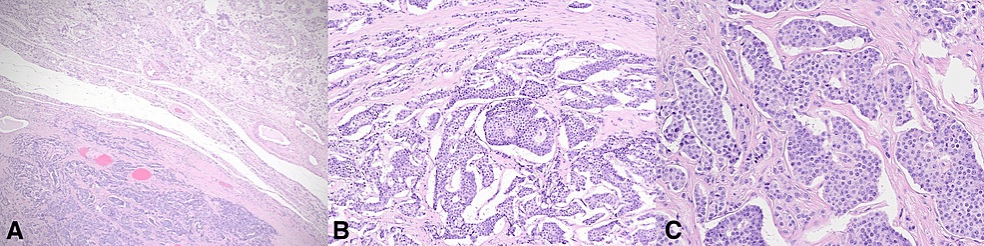
Histopathological examination of the specimen at low-power view showing (A) a well-defined neoplastic tumor with normal testicular tissue. At intermediate-power view, (B) the neoplastic cells are arranged in nests, cribriform, and micro-glandular patterns. At high-power view, (C) the tumor cells have round monotonous cells with coarse, salt and pepper nuclear chromatin. No atypia, necrosis, or mitosis can be seen.

**Figure 3:**
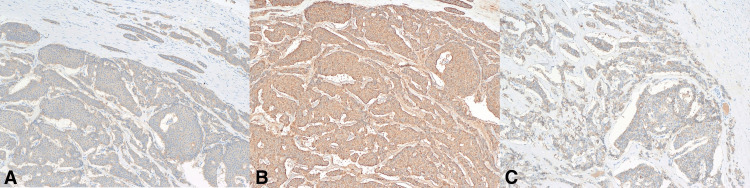
Immunohistochemical examination of the specimen showing positive staining for synaptophysin (A), chromogranin A (B), and CD56 (C).

Microscopic examination at a low-power view showed a well-defined neoplastic tumor with normal testicular tissue (Figure 2a). At an intermediate-power view, the tumor cells formed nests, cribriform, and micro-glandular patterns (Figure 2b). The tumor cells have had round, monotonous cells with coarse, salt and pepper nuclear chromatin. There was no atypia, necrosis, or mitosis (Figure 2c), and no evidence of germ cell tumors, germ cell neoplasia in situ, or teratoma components. A panel of immunohistochemical studies was requested, which showed that the tumor cells were positive for synaptophysin (Figure 3a), chromogranin A (Figure 3b), and CD56 (Figure 3c). The Ki67 index was less than 1%. Other immunostains, including placental alkaline phosphatase, C-KIT, D2-40, b-HCG, and alpha-fetoprotein (AFP) were negative. These findings were consistent with a testicular neuroendocrine tumor.

There was no evidence of metastatic neuroendocrine tumor either in the chest, abdomen, or pelvic computed tomography scan. Additionally, there was no evidence of specific hypermetabolic abnormality over the examined body volume on positron emission tomography-computed tomography (whole body).

## Discussion

Neuroendocrine tumors originate from neuroendocrine cells that spread throughout the body. Commonly these tumors are called carcinoid tumors [3]. They secrete different kinds of peptides that may cause characteristic hormonal syndromes [4]. In a paper published by the National Cancer Institute, Surveillance Epidemiology and End Results program, in 11,427 carcinoid patients treated from 1973 to1997, about 54% of the tumors were located at the gastrointestinal tract, with the duodenum as the most common site [5]. Testicular neuroendocrine tumors are exceedingly rare, representing less than 1% of all testicular tumors, and the first primary testicular carcinoid was described by Simon et al. in 1954 [1,6]. A total of 135 cases of testicular neuroendocrine tumors have been reported in the literature [1,2,7,8]. The origin of a primary testicular neuroendocrine tumor is controversial. Some authors believe that the origin of the testicular neuroendocrine tumor is a differentiation of the pluripotential germ cell to argentaffin-like cells [9]. They are divided into three categories: pure primary testicular neuroendocrine tumors, metastatic neuroendocrine tumors, and neuroendocrine tumors associated with teratoma [2]. Most commonly they are detected incidentally. However, the most common complaint is a painless mass with an enlarged testis [10]. Fewer than 8% of the cases are associated with carcinoid syndrome [2]. Once the diagnosis is confirmed, metastasis should be excluded by a CT scan of the abdomen, pelvis, and chest and baseline 24-hour urinary 5-HIAA in addition to serum serotonin [10]. Radical orchiectomy remains the mainstay treatment of localized testicular neuroendocrine tumors, though chemotherapy and radiotherapy have been found to have limited effects in a few select cases [5]. Generally, the tumor behaves in an indolent fashion [10]. While those with large size, high-grade morphology, and associated with carcinoid syndrome are usually more aggressive [2]. The testicular neuroendocrine tumor associated with a teratomatous component seems to have a better prognosis than the pure form [2]. Similar cases published in the literature are described in Table 1.

**Table 1:** Summary of previously published studies of testicular neuroendocrine tumors. TNET = testicular neuroendocrine tumors

Study	Tumor type	Number of cases	Case year	Age	Tumor site	Tumor size (mm)	Mean size (mm)	Immunohistochemistry study
Amine et al. [2]	Pure primary TNET	101	1930–2015	10–83 (average: 39 years)	Left testis: 50; right testis: 44; bilateral: 2; remaining not specified.	3–110	41.75	Synaptophysin: done in 45 with a positive result (100%). Chromogranin A: done in 59 with a positive result (100%)
	TNET-associated teratoma	22				10–80	36.91	
	Secondary TNET	9				5–60	22.13	
Manna et al. [8]	Pure primary TNET	1	2020	31 years	Left testis	600	NA	Synaptophysin: positive. Chromogranin A: positive. CD56: positive
Wonglhow et al. [7]	TNET-associated teratoma	1	2022	37 years	Right testis	800	NA	Synaptophysin: positive. Chromogranin A: positive. CD56: positive
Xiao et al. [1]	Pure primary TNET	1	2022	24 years	Right testis	110	NA	Synaptophysin: positive. Chromogranin A: positive. CD56: positive
Present case	Pure primary TNET	1	2023	47 years	Right testis	350	NA	Synaptophysin: positive. Chromogranin A: positive. CD56: positive

Li ﻿et al. (2014) [11] reported a case of a 46-year-old male who was referred after a magnetic resonance imaging study showed an incidental finding of a 2 cm right testicular mass. The serological study showed elevated AFP (4.49 ng/mL), β-HCG (0.08 mIU/mL), and lactate dehydrogenase (197 U/L). Scrotal sonography showed a heterogeneous echoic mass in the right testicle measuring 1.9 cm, with increased vascularity and a calcified core. The patient underwent an uneventful right radical orchiectomy afterward. Microscopic examination showed monotonous cells with oval nuclei, salt and pepper chromatin, and granular cytoplasm. Neuroendocrine markers (synaptophysin, CD56, and chromogranin) were positive in the tumor cells, while alpha-inhibin was negative (to rule out other germ cell tumors). No teratomatous component ‎was found. The patient was followed up with CT, scrotal sonography, and measurement of urinary 5-HIAA. After three years of close follow-up, there was no evidence of disease recurrence.

Thomas and Jones (2004) [10] reported a case of a 41-year-old male who was to have elective sterilization. Upon preoperative evaluation, the physical examination was unremarkable, and there was no history of any chronic medical condition. Routinely, before the procedure, a warm towel is to be used to reheat the scrotum to help find the vas deferens. However, after reheating, the right testicle was significantly enlarged which was not appreciated upon preoperative clinical examination. A scrotal ultrasound was performed postoperatively, and a hypoechoic lesion in the upper aspect of the right testicle measuring 3 cm was found. Unexpectedly, AFP and β-HCG levels were not elevated. The patient had an uneventful right radical orchiectomy afterward. Microscopic examination showed tumor cells with neuroendocrine differentiation along with a teratomatous component‎. Neuroendocrine markers (synaptophysin and chromogranin) were positive in the tumor cells. CT (for chest, abdomen, and pelvis), urinary 5-HIAA level, and serum serotonin level were requested to complete the metastatic workup but were all unremarkable.

## Conclusions

Well-differentiated neuroendocrine tumor of the testis is an extremely rare neoplasm with a good prognosis. The diagnosis of a primary neuroendocrine tumor of the testis is based on histologic examination, immunohistochemistry study, and the absence of other primary involvement. The gold standard of treatment is radical orchiectomy with close follow-up to exclude metastasis.

## References

[1] Xiao T, Luo LH, Guo LF, Wang LQ, Feng L (2022). Primary testicular neuroendocrine tumor with liver lymph node metastasis: a case report and review of the literature. World J Clin Cases.

[2] Amine MM, Mohamed B, Mourad H (2017). Neuroendocrine testicular tumors: a systematic review and meta-analysis. Curr Urol.

[3] Moch H, Amin MB, Berney DM (2022). The 2022 World Health Organization classification of tumours of the urinary system and male genital organs-part A: renal, penile, and testicular tumours. Eur Urol.

[4] Yao JC, Hassan M, Phan A (2008). One hundred years after "carcinoid": epidemiology of and prognostic factors for neuroendocrine tumors in 35,825 cases in the United States. J Clin Oncol.

[5] Maggard MA, O'Connell JB, Ko CY (2004). Updated population-based review of carcinoid tumors. Ann Surg.

[6] Simon HB, McDonald JR, Culp OS (1954). Argentaffin tumor (carcinoid) occurring in a benign cystic teratoma of the testicle. J Urol.

[7] Wonglhow J, Sunpaweravong P, Sathitruangsak C, Kanjanapradit K, Dechaphunkul A (2022). Metastatic primary testicular neuroendocrine carcinoma associated with somatic malignant transformation of teratoma: a rare case report. Case Rep Oncol.

[8] Manna S, Narayana SM, Premalata CS (2020). Primary neuroendocrine tumor of the testis masquerading as germ cell tumor - a case report. Eur J Med Health Sci.

[9] Park SB, Kim JK, Cho KS (2006). Imaging findings of a primary bilateral testicular carcinoid tumor associated with carcinoid syndrome. J Ultrasound Med.

[10] Thomas JC, Jones JS (2004). Primary carcinoid tumor of the testis found at the time of elective sterilization. J Androl.

[11] Li HK, Huang EY, Lin AT, Chen KK (2014). Testicular carcinoid tumor: a case report and literature review. Urol Sci.

